# Regulation of Dynamic Protein S-Acylation

**DOI:** 10.3389/fmolb.2021.656440

**Published:** 2021-04-26

**Authors:** Jessica J. Chen, Ying Fan, Darren Boehning

**Affiliations:** Department of Biomedical Sciences, Cooper Medical School of Rowan University, Camden, NJ, United States

**Keywords:** S-acylation, DHHC enzymes, APT, ABHD, cell signaling

## Abstract

Protein S-acylation is the reversible addition of fatty acids to the cysteine residues of target proteins. It regulates multiple aspects of protein function, including the localization to membranes, intracellular trafficking, protein interactions, protein stability, and protein conformation. This process is regulated by palmitoyl acyltransferases that have the conserved amino acid sequence DHHC at their active site. Although they have conserved catalytic cores, DHHC enzymes vary in their protein substrate selection, lipid substrate preference, and regulatory mechanisms. Alterations in DHHC enzyme function are associated with many human diseases, including cancers and neurological conditions. The removal of fatty acids from acylated cysteine residues is catalyzed by acyl protein thioesterases. Notably, S-acylation is now known to be a highly dynamic process, and plays crucial roles in signaling transduction in various cell types. In this review, we will explore the recent findings on protein S-acylation, the enzymatic regulation of this process, and discuss examples of dynamic S-acylation.

## Introduction

Protein lipidation is the co-translational or post-translational covalent addition of a variety of lipids to target proteins. Lipidation of proteins plays an important role in the regulation of cellular functions by affecting protein activity, localization, and stability (Wang and Casey, 2016; Zurzolo and Simons, 2016; Jiang et al., 2018). At least five different lipid classes are enzymatically attached to proteins, including glycosylphosphatidylinositol (GPI) anchors, isoprenoids, sterols, phospholipids and fatty acids. The GPI anchor attaches proteins to the exterior surface of the cell through a glycolipid containing phosphatidylinositol. The attachment of a GPI anchor occurs at the carboxy-terminus and is mediated by more than 20 enzymes (Resh, 2013; Masuishi et al., 2016). Protein *O*-cholesterylation is the attachment of a sterol group to the C-terminus of a protein. This modification is especially common in the mammalian Hedgehog protein family, which are secreted proteins that have signaling function in regulating many tissues and structures in development (Nüsslein-Volhard and Wieschaus, 1980; Porter et al., 1996). N-Myristoylation is an irreversible covalent attachment of a 14-carbon saturated fatty acid to Gly residues on the N-terminus of proteins (Carr et al., 1982). Myristoylation occurs after the methionine aminopeptidase processing of the initiator methionine exposes the Gly residue at position 2 (Wilcox et al., 1987). Many proteins involved in signaling pathways, apoptosis, cancer, and viral replication are known to be myristoylated (Thinon et al., 2014). Protein prenylation adds the 15-carbon farnesyl motif or the 20-carbon geranylgeranyl motif to target proteins (Kamiya et al., 1978). In general, prenylation is an enzymatically mediated multi-step process that adds hydrophobic prenyl moieties to the C-terminal cysteines of substrate proteins. Three different protein prenyltransferases—farnesyltransferase and geranylgeranyltransferases I and II—catalyze protein prenylation. Prenylation is a clinically important regulator of Ras protein function (Jackson et al., 1990).

There are many detailed reviews outlining the consensus sequences, enzymatic regulators, and functional outcomes of different classes of lipid post-translational modification (Resh, 2013; Chen and Boehning, 2017; Chen et al., 2018; Jiang et al., 2018). For this review, we will be focusing on protein acylation. Fatty acyl groups such as palmitate can be added to glycine or lysine (N-acylation), serine or threonine (O-acylation), and cysteines (S-acylation) (Kleuss and Krause, 2003; Buglino and Resh, 2008; Zou et al., 2011; Jing et al., 2017). For the rest of this review, we specifically discuss S-acylation, the covalent linkage of various fatty acids (14–20 carbons) *via* a thioester bond to the cysteine residues of substrate proteins. While the majority of the lipid modifications to proteins are irreversible, S-acylation is reversible and can be highly dynamic. When looking at S-acylation turnover on a proteomic scale, the majority of proteins have minimal turnover during the course of several hours, suggesting they are stably modified (Martin et al., 2012). However, a subset of proteins are dynamically S-acylated and they are implicated in key cellular events such as cell growth. One of the most well-known lipids involved in S-acylation is the 16-carbon saturated palmitic acid. However, studies have shown that various saturated and unsaturated fatty acids, including oleic acid (Liang et al., 2001; Montigny et al., 2014; Thinon et al., 2016), steric acid (Kordyukova et al., 2008; Senyilmaz et al., 2015; Thinon et al., 2016), and arachidonic acid (Liang et al., 2001), can be incorporated onto cysteine residues in substrate proteins. In human platelets, 74% of the fatty acids linked to proteins *via* thioester bonds are palmitic acid, followed by about 22% stearic acid and 4% oleic acid (Muszbek et al., 1999). Studies using bovine retina, heart, and liver proteins also showed a variety of thioester-linked fatty acids, with palmitic and stearic acids being the top two most abundant lipid modifications (DeMar Jr and Anderson, 1997).

Perhaps not surprisingly, the profile of fatty acids conjugated to S-acylated proteins depends on the exogenous supply of fatty acids (Muszbek et al., 1999; Webb et al., 2000), For example, incubation of human platelets with exogenous palmitate and stearate increased the amount of proteins palmitoylated and stearylated by 26% and 30%, respectively (Muszbek et al., 1999). However, how S-acylation lipid profiles are regulated endogenously remains unclear. Additionally, the lipid species added can vary from cysteine to cysteine on the same protein (Brett et al., 2014). For example, the Influenza A virus protein hemagglutinin has three S-acylated cysteines, one of which is exclusively modified with the 18-carbon stearic acid (Kordyukova et al., 2008). While majority of the studies focus on palmitoylation, it is worth noting that incorporation of other fatty acids can lead to different functional outcomes (Liang et al., 2001; Senyilmaz et al., 2015). The prevalence or functional significance of fatty acids other than palmitic acid on majority of the S-acylated proteins is not fully understood.

To date, there is no universal consensus sequence for S-acylation sites (Salaun et al., 2010). However, there are some recurring patterns in specific classes of proteins. Src family kinases (Shenoy-Scaria et al., 1993; Koegl et al., 1994) and some of the heterotrimeric G proteins (Clapham and Neer, 1993; Linder et al., 1993) are acylated at N-terminal cysteines adjacent to the myristoylation site. Ras proteins have acylated cysteines adjacent to the C-terminal prenylation sites (Hancock et al., 1989). Many transmembrane proteins, such as claudin proteins found at tight-junctions (Rodenburg et al., 2017), are acylated at juxtamembrane positions. It is worth noting that we are just starting to appreciate the prevalence of S-acylation—approximately 20% of the mammalian proteome is predicted to be acylated (Blanc et al., 2019). One of the most widely used S-acylation databases is SwissPalm (Blanc et al., 2015). The recently updated SwissPalm 2 includes data from 38 palmitoyl-proteomic studies across 17 species (Blanc et al., 2019).

Recently, there is growing interest in understanding the enzymes regulating the highly dynamic S-acylation cycle on signaling proteins. As noted above, palmitoyl acyltransferases (PATs) catalyze the addition of fatty acyl groups (Lobo et al., 2002; Roth et al., 2002; Mitchell et al., 2006) while acyl proteins thioesterases (APTs) (Duncan and Gilman, 1998; Tomatis et al., 2010) and the α/β hydrolase domain (ABHD) proteins (Lin and Conibear, 2015) are responsible for the removal of Acyl groups. In this review, we will discuss recent developments in understanding both classes of enzymes. We will also summarize the functions of protein S-acylation by providing examples from the literature. Additionally, we will describe the methods used to study S-acylation and discuss their pros and cons. Finally, we will provide three examples of dynamic acylation in different cell types and the enzymatic machineries that tightly regulate them.

## Functional Outcomes of S-Acylation

### Membrane Localization

Many membrane-associated proteins without a transmembrane domain require the conjugation of fatty acids for targeting to the membrane surface. Interestingly, different types of lipid modifications often exist on the same protein to achieve stable membrane attachment. For example, multiple members of the heterotrimeric G protein family (Clapham and Neer, 1993; Linder et al., 1993) and the Src family kinases (Shenoy-Scaria et al., 1993; Koegl et al., 1994) have other forms of lipidation in addition to S-acylation. Prenylation and myristoylation of proteins lead to transient and weak binding to lipid bilayers, and therefore are often not sufficient for reliable membrane targeting (Bhatnagar and Gordon, 1997). Fatty acids attached *via* S-acylation groups often leads to a more thermodynamically and kinetically stable binding to the membrane (Shahinian and Silvius, 1995). Since all known PATs are integral membrane proteins, it is proposed that other lipid modifications and/or intrinsic membrane affinity of peripheral protein substrates bring them in proximity to the PATs to facilitate S-acylation (Chamberlain and Shipston, 2015). For example, mutations of the prenylation sites in Ras proteins lead to decreased acylation (Hancock et al., 1989).

Lipid bilayers contain microdomains that are enriched in cholesterol and sphingolipids, commonly known as lipid rafts or liquid-ordered domains [reviewed in Levental et al. (2020)]. These microdomains have structural and functional importance in signaling transduction, endocytosis, and other cellular events (Simons and Toomre, 2000; Parton and Richards, 2003). We will refer to these lipid microdomains for the remainder of this review as lipid rafts. S-acylated proteins are known to be enriched in lipid rafts because long saturated acyl chains favor partitioning into the highly ordered environment created by cholesterol and sphingolipids [reviewed in Levental et al. (2010)]. One example is the influenza viral membrane protein hemagglutinin. S-acylation is important for its association with lipid rafts (Melkonian et al., 1999), which subsequently facilitates viral replication (Zurcher et al., 1994) and viral fusion (Naeve and Williams, 1990). Non-acylated hemagglutinin fails to cluster in lipid rafts, which later become the fusion sites (Takeda et al., 2003). Similarly, multiple members of the T cell receptor complex (Rodgers et al., 1994; Arni et al., 1996; Parolini et al., 1996; Harder and Kuhn, 2000) and neuronal synaptic proteins (Topinka and Bredt, 1998; Yamazaki et al., 2001; Hering et al., 2003) require S-acylation for their localization to lipid rafts.

S-acylation is neither necessary nor sufficient for lipid raft localization. For example, the anthrax toxin receptor tumor endothelial marker 8 (TEM8) is acylated on several cysteine residues, and acylation functions to exclude the protein from rafts (Abrami et al., 2006). Caveolin-1 is acylated on three cysteines, but these modifications are not necessary for its localization to caveolae, a specialized lipid raft (Dietzen et al., 1995). Members of the Src family kinases are excluded from lipid rafts when S-acylated with polyunsaturated fatty acids (Liang et al., 2001). One of the patterns that are associated with lipid raft recruitment of target proteins is dual palmitoylation in addition to myristoylation or prenylation. In the case of single-pass transmembrane proteins, the length and surface area of the transmembrane domain and their S-acylation state can be used to reliably predict their lipid raft affinity (Lorent et al., 2017). Another factor that was shown to regulate the enrichment of S-acylated proteins in membrane domains is membrane curvature (Larsen et al., 2015). However, many other factors, including the type of lipid added and its interaction with other proteins, contribute to the association of acylated proteins with lipid rafts. Therefore, the specific effects of S-acylation on membrane partitioning should be analyzed in each individual case.

### Trafficking

Protein trafficking is a highly regulated process to ensure that proteins are targeted to the correct cellular location for function (Mellman and Nelson, 2008). Many proteins that are transported between the Golgi, plasma membrane, and the endosomal recycling system require S-acylation for trafficking to their designated location [reviewed in Diaz-Rohrer et al. (2014)]. One example is the soluble N-ethylmaleimide-sensitive factor attachment protein receptors (SNAREs), which are responsible for fusion of membranes at different cellular locations (Jahn and Scheller, 2006). The synaptic-associated protein of 25 kDa (SNAP25) is one of the SNARE proteins that regulate synaptic vesicle exocytosis, and it has four acylation sites (Gonzalo et al., 1999). In addition to membrane attachment, the acylated cysteines determine the distribution of SNAP25 among the trans-Golgi network (TGN), plasma membrane, and the recycling endosomes (Greaves and Chamberlain, 2011). Specifically, decreasing the number of cysteines leads to SNAP25 accumulation in the TGN and recycling endosomes. Interestingly, increasing the number of SNAP25 acylation sites enhances their association with cholesterol-rich membranes *in vitro* (Salaün et al., 2005). It is proposed that vesicle budding occurs at lipid-raft like regions in the TGN during anterograde trafficking and recycling endosome membranes when recycling proteins back to the plasma membrane (Patterson et al., 2008; Diaz-Rohrer et al., 2014). Therefore, SNAP25 acylation laterally segregate the protein in membrane subdomains to prepare for their exit from the TGN or recycling endosomes and the subsequent delivery to the plasma membrane.

In polarized cells where proteins are delivered to specialized subcellular compartments such as axons and dendrites in neurons, S-acylation can target proteins to their destinations. One example is the glutamic acid decarboxylase 65 (GAD65), which synthesizes the inhibitory neurotransmitter gamma-amino butyric acid (GABA) (Baekkeskov et al., 1990). GAD65 is palmitoylated at two cysteines in the N-terminal domain, and the palmitoylation state does not affect its membrane anchoring at the Golgi apparatus (Shi et al., 1994). Instead, S-acylation of GAD65 is important for its trafficking to the presynaptic sites of axons (Kanaani et al., 2002). Specifically, S-acylation excluded GAD65 from the *cis-*Golgi face and moved them to the trans-Golgi membrane (Kanaani et al., 2008). S-acylation is also required for sorting GAD65 into giant axonal vesicles at TGN where it co-localizes with Rab5a, a GTPase that regulates vesicle motility on microtubules (Kanaani et al., 2004). The mechanisms by which S-acylated GAD65 interacts with Rab5a or other components of giant axonal vesicles remain to be explored. Interestingly, de-acylated GAD65 can be recycled back to the Golgi *via* a non-vesicular pathway, suggesting dynamic regulation at the axonal terminal (Kanaani et al., 2008).

Transmembrane proteins such as ion channels (Hayashi et al., 2005; Hayashi et al., 2009) and transporters (Ren et al., 2015; Du et al., 2017), as well as peripheral proteins such as Ras GTPases (Hancock et al., 1989) rely on S-acylation for localizing to different subcellular locations. Majority of the PAT enzymes that S-acylate target proteins are localized to the Golgi, where newly synthesized proteins are sorted and distributed. Therefore, it is not surprising that S-acylation serves as an important signal for protein trafficking. For example, S-acylation of anterograde cargo proteins such as vesicular stomatitis virus G (VSVG) protein and transferrin receptors facilitates their transport from the *cis-*to the *trans-*Golgi (Ernst et al., 2018). However, several PATs are also found at the plasma membrane and ER, where local acylation cycles regulate protein functions (Philippe and Jenkins, 2019). The extent by which such local acylation affect protein trafficking is a topic that is still currently under investigation.

### Protein Interactions

S-acylation of proteins can regulate their interactions with other proteins and subsequently affect their function. One example is the endoplasmic reticulum (ER)-localized chaperone calnexin, which facilitates the folding of glycoproteins (David et al., 1993). Calnexin is a transmembrane protein with a very large luminal domain and a short cytoplasmic tail. Calnexin has two acylated cysteines at juxtamembrane positions, and acylation is predicted to alter its cytosolic tail orientation (Lakkaraju et al., 2012). S-acylation enables calnexin to interact with the ribosome-translocon complex and the actin cytoskeleton, further stabilizing the supercomplex to ensure chaperone access to protein substrates as they enter the ER lumen (Lakkaraju et al., 2012).

S-acylation of the G-protein coupled receptor β2 adrenergic receptor (β2AR) also regulates its protein interactions. Activation of β2AR leads to production of cyclic adenosine monophosphate (cAMP). After activation, β-arrestin binds to the receptor to facilitate receptor internalization and termination of the signal (Dzimiri, 1999). S-acylation of β2AR at Cys 341 enhances its interaction with phosphodiesterase 4D and β-arrestin 2 (Liu et al., 2012). However, the structural properties that are altered by acylation to favor such interactions remain unclear. The β2AR can also be acylated at Cys 265, which stabilizes the protein at the plasma membrane after sustained receptor activation (Adachi et al., 2016). Interestingly, acylation at this site is mediated by the Golgi-specific DHHC9/14/18. Inhibiting APT1 blocked stimulus-dependent deacylation at Cys 265, suggesting that stimulus-dependent regulation of APT1 activity at the plasma membrane is a regulator of β2AR signaling.

In neurons, acylation of postsynaptic density protein-95 (PSD-95) is crucial for its interaction with cyclin-dependent kinase-like 5 (CDKL-5), which promotes neurite growth and stabilizes synapses (Zhu et al., 2013). It has been reported that acylation of PSD-95 alters the conformation of the protein from a compact to extended conformation (Jeyifous et al., 2016). Therefore, it is possible that the extended conformation of PSD-95 exposes the binding site with CDKL-5. The ability of acylation to regulate protein structure and either directly or through allosteric changes influence protein binding surfaces is now becoming increasingly appreciated, and will be discussed in more detail below.

### Protein Half-Life and Stability

Protein S-acylation can directly influence substrate stability since the absence of S-acylation can lead to decreased half-life of the protein. TBC1 domain family member 3 (TBC1D3) is a plasma membrane protein overexpressed in several human cancers (Pei et al., 2002; Wu et al., 2011). TBC1D3 is acylated on Cys residues 318 and 325 (Kong et al., 2013). When S-acylation of TBC1D3 is inhibited pharmacologically or through mutagenesis, protein half-life is greatly reduced due to increased degradation. The degradation of TBC1D3 is mediated through ubiquitination by E3 ubiquitin ligase cullin-7 (CUL7) and subsequent proteasomal degradation (Kong et al., 2012; Kong et al., 2013). S-acylation deficient TBC1D3 seems to be a preferred substrate for CUL7 (Kong et al., 2013). As such, deacylation of TBC1D3 leads to ubiquitination and proteasomal degradation. The possible protective mechanism by which S-acylation prevents TBC1D3 binding to CUL7 is by sequestering the protein to lipid rafts or by altering its conformation.

Another example of S-acylation regulating protein stability on the plasma membrane is the Fas death receptor (FasR). FasR is a member of the tumor necrosis factor receptor superfamily and represents a canonical death receptor (Ashkenazi and Dixit, 1998). After FasR is activated by its ligand, it starts a cascade of signaling which leads to activation of caspases and eventually to cell death (Kischkel et al., 1995). Fas receptor S-acylation occurs on an intracellular cysteine next to the transmembrane domain, and this modification is necessary to trigger cell death (Chakrabandhu et al., 2007; Feig et al., 2007). FasR S-acylation stabilizes it at the plasma membrane and prevents premature degradation of the receptor. The S-acylation deficient mutant of Fas leads to lysosomal degradation of the protein. FasR activation leads to receptor oligomerization and concentration in lipid rafts. S-acylation of FasR facilitates recruitment to lipid rafts, and subsequent stability of the oligomerized receptor and associated death-induced signaling complex (Rossin et al., 2015).

The unique reversibility of S-acylation allows the cycling of proteins from an S-acylated stable form to deacylated degradation-susceptible protein. In addition to the two examples mentioned above, many other proteins require acylation to prevent their degradation. For example, acylation of C–C motif of chemokine receptor 5 (CCR5) prevents it from entering lysosomal degradation pathways (Percherancier et al., 2001). Another example is the acylation of the yeast SNARE protein Tlg1, which protects it from ubiquitination and degradation (Valdez-Taubas and Pelham, 2005). We are just starting to understand the importance of S-acylation in maintaining protein homeostasis through various protein degradation pathways. Future studies should focus on acylation as a key component of the quality control network and explore its potential roles in disease-associated protein aggregation.

### Protein Conformation

Conformational changes are an integral part of protein function, regulating protein and ligand binding, localization, and stability. By studying a synthetic hydrophobic peptide which is reconstituted into liposomes, it was shown that S-acylation can change the orientation of transmembrane domains (Joseph and Nagaraj, 1995), which is consistent with modeling studies (Morozova and Weiss, 2010). This is of importance since most acylation sites in transmembrane proteins are adjacent to the membrane (Charollais and Van Der Goot, 2009; Morozova and Weiss, 2010). One particularly dramatic example on how S-acylation can regulate membrane protein conformation is exemplified by lipoprotein receptor related protein 6 (LRP6). LRP6 is a type I membrane protein synthesized with an N-terminal signal sequence which targets it to the ER (Hsieh et al., 2003). LRP6 maturation and exit from the ER involves S-acylation to ensure correct folding and orientation of the transmembrane domain. S-acylation of Cys-1394 and Cys-1399 changes the length of the hydrophobic stretch of the transmembrane domain and thus alleviating the hydrophobic mismatch with the thickness of the ER membrane. Mutation of the S-acylation sites leads to retention of LRP6 in the ER. Newly synthesized LRP6 is also ubiquitinated. Instead of being degraded, ubiquitinated LRP6 is recognized by cytosolic chaperones to promote folding, and once folded it is deubiquitinated (Feldman and van der Goot, 2009). Once properly folded and S-acylated, LRP6 exits the ER and transits through the Golgi before arriving at the plasma membrane where its signaling role takes place (Abrami et al., 2008). The current model of LRP6 maturation is sequential ubiquitination, folding, and S-acylation before the mature protein can exit the ER (Perrody et al., 2016).

Another example how S-acylation regulates protein conformation is the MHV-A59 murine coronavirus spike protein that executes viral cell entry functions. Cell entry requires major conformational changes of the spike protein (Zelus et al., 2003). The spike protein cytosolic tail from several coronaviruses has a cysteine-rich tail adjacent to the cytosolic surface of the transmembrane domain. The tail of the Mouse Hepatitis Virus (MHV) spike protein is extensively S-acylated (Thorp et al., 2006). In spike mutants lacking all three S-acylated cysteine residues, the spike protein remained in the transitional folding state almost 10 times longer than the wild type protein (Shulla and Gallagher, 2009). Moreover, acylation-deficient spike mutants failed to incorporate into secreted virions efficiently, and those few that managed to get into virions cannot support virus-cell entry and fusion efficiently because they are slow to refold into post fusion forms. S-acylation is required for virus spike proteins to unfold into the pre hairpin state at cell surfaces, immediately after binding cell surface receptors. For those viruses without S-acylated spike proteins, this pre hairpin structure remains as viruses enter endosomes, and are then cleaved away by endosomal proteases. These findings indicate S-acylation of the cytosolic tail can anchor spike protein onto virion membranes. S-acylation is required for maintaining metastable prefusion spike conformations and for the progression through conformational changes during infection (Shulla and Gallagher, 2009). Other coronavirus spike proteins are subject to acylation of the cytosolic tail, including SARS-CoV (Petit et al., 2007). Similar to MHV, SARS-CoV spike protein acylation is critical for S-mediated cell fusion. SARS-CoV-2 also has a cysteine-rich cytosolic domain, however whether this domain is acylated has not been investigated. Since low doses of acylation inhibitors such as 2-bromopalmitate profoundly inhibit coronavirus infectivity (Thorp et al., 2006), this is an important and timely area for further study.

Conformational changes in proteins can be influenced by cell signaling events. The emerging roles of dynamic S-acylation in eukaryotic cells highlight the potentially important role of this modification in regulating protein conformation in response to cellular stimuli. It will be of particular interest to study the kinetics of S-acylation in relation to subsequent molecular and cellular events. For example, by using fluorescence resonance energy transfer (FRET) in live cells, it was recently found that the dynamic palmitoylation of sodium-calcium exchanger 1 (NCX1) leads to rearrangement of the f-loop region and regulate its dimerization (Gök et al., 2020). These acylation-induced changes are key in recruiting the exchange inhibitory peptide and mediating Ca^2+^ influx. Another key regulator of action potentials, the voltage-gated sodium channel Na_v_1.5, also requires S-acylation for its function (Pei et al., 2016). It has been shown that S-acylation-induced conformational changes are important for its activation and the excitability of cardiac cells.

## Methods of Detecting S-Acylation

### Radiolabeling

Various techniques have been developed to detect protein S-acylation. Each technical approach has their own advantages, limits, and applications (Table 1). The earliest method developed was metabolic labeling with radioactive isotope-labeled palmitic acids, followed by immunoprecipitation of selected target protein and using autoradiography to detect the labeled fatty acid (Schlesinger et al., 1980; Bizzozero, 1995; Caron, 1997). Metabolic labeling with radiolabeled palmitate has been used to effectively identify S-acylated proteins and detect the residence half-life of either palmitate on a specific protein or a palmitate turnover. ^14^C-, ^3^H-, and ^125^I-labeled palmitates were all used in various studies, but ^3^H-palmitate is the most common due to its low cost, safety, and wide availability (Ducker et al., 2006; Kenworthy, 2006; Veit et al., 2008). Despite its wide application in many studies for years, the disadvantages of radiolabeling include low sensitivity, long exposure time (days to weeks), and the hazards associated with the use and disposal of radioactive material.

**TABLE 1:** Comparison of different methods detecting S-acylation. Selected methods are listed above with their targets, advantages, and disadvantages.

Method	Target	Advantages	Disadvantages
Radiolabeling	Cells, purified protein	—	1. Long exposure time (days to weeks)
			2. Use of radioactive material
			3. Only detect protein palmitoylation
Click chemistry based labeling	Cells	1. High detection sensitivity	1. Only detect proteins that are palmitoylated during experiment
		2. Convenient experiment procedure	2. May interfere metabolism of cells
		3. High specificity	3. Efficiency of probes incorporated onto substrates varies
		4. Allow protein pull down	
		5. Allow to study the dynamics of protein S-acylation	
Acyl-biotin exchange based assay	Cells, tissues	1. Higher specificity	1. Possible false-positive results
		2. Detect S-acylation in a steady level	2. Require time course to study dynamic S-acylation
		3. Enable mass spectrometry-based analysis	
		4. Allow protein pull down	
		5. Show the relative amount of protein in each acylation state	
Spectrum counting	Cells	1. Simpler sample preparation	Requires samples prepared and analyzed separately
		2. Direct comparison of multiple samples	
Stable isotope-labeled peptide with mass spectrometry	Cells	1. Minimizes quantitative errors	Some S-acylated peptides cannot be quantified (SILAC)
		2. Increased reproducibility, accuracy, and sensitivity	
		3. Reduce the spectral complexity	
		4. Double verification for the identified peptides	

### Click Chemistry-Based Labeling

**FIGURE 1:**
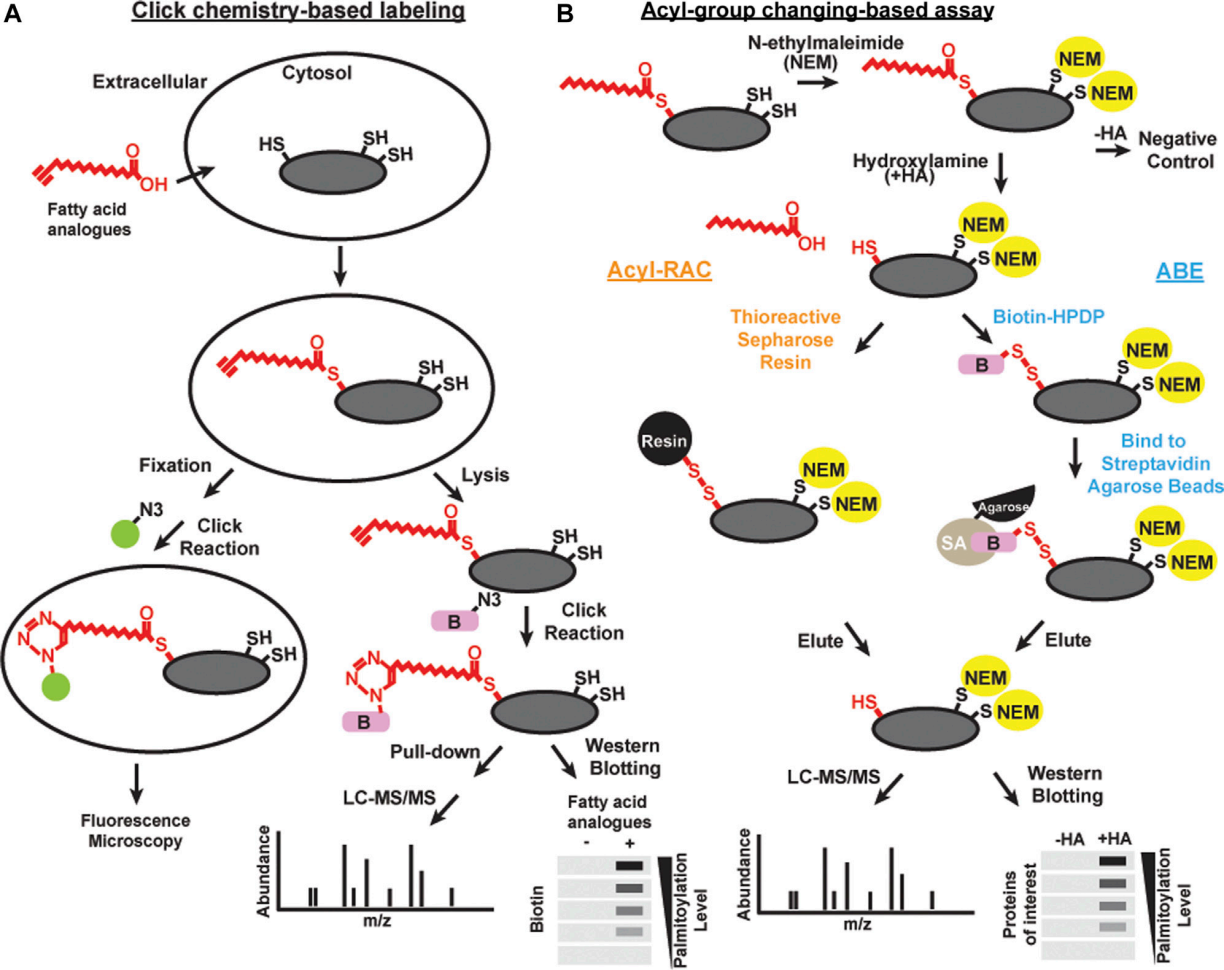
Schematics of methods of detecting acylation Click chemistry-based labeling **(A)** and acyl-group changing-based assay **(B)** can be combined with various detection methods, including microscopy, liqued chromatography and tandem mass spectrometry (LC-MS/MS), and western blotting, to detect S-acylated proteins.

“Click chemistry” is a general term to describe simple, high yield, chemical reactions (Kolb et al., 2001). One click chemistry reaction is the copper-catalyzed cycloaddition reaction between azide and alkyne groups (Kolb and Sharpless, 2003). This reaction and related cycloaddition reactions are useful to interrogate biological systems because alkynes and azides are biologically inert [bioorthogonal; Devaraj (2018)]. Click chemistry with alkyne-conjugated lipids was developed to facilitate the detection and purification of acylated proteins (Figure 1A). Synthetic fatty acid analogues with alkyne groups at the terminal end furthest away from the carboxyl group (ω-position) are incorporated into live cells by metabolic labeling similar to the radiolabeling approach (Hannoush and Sun, 2010). Cells are then lyzed or fixed, and the synthetic lipids incorporated into proteins are subjected to a copper-catalyzed reaction with azide-conjugated probes such as fluorophores or biotin (Wang et al., 2003). Click chemistry-based probes are particularly useful in studies examining the selectivity of incorporation of different fatty acid groups (Greaves et al., 2017).

Brief exposure of alkyne-labeled lipids to live cells permits investigation of the acylation dynamics of newly acylated proteins similar to a classic “pulse-chase” experiment done with radioactivity (Zhang et al., 2010; Martin et al., 2011). There are some caveats to using this approach. 17-Octadecynoic acid is an alkyne probe frequently used to label S-acylated cysteines. However, it can also be processed with low efficiency at N-myristoylation sites (Martin and Cravatt, 2009). In addition, proteins with a glycosylphosphatidylinositol (GPI) anchor can also be labeled with palmitic acid probes (Jones et al., 2012; Wright et al., 2014). Click chemistry has led to many important discoveries in protein S-acylation, but the efficiency of incorporating alkyne conjugated probes onto substrates are different, thus is not the best choice for unbiased proteomic studies in native tissues. Finally, feeding exogenous lipids to cells leads to significant direct and indirect effects on cellular physiology, and as such both radiolabeling and click chemistry approaches need to be interpreted with caution.

### Acyl-Group Exchange-Based Assay

Acyl-biotin exchange (ABE) is a method based on the *in vitro* exchange of thioester-linked lipids attached to cysteines to derivatives of biotin (Figure 1B). There are three main steps: 1) irreversible blocking of all the free cysteines by alkylating reagents, often with N-ethylmaleimide or S-Methyl methanethiosulfonate; 2) selective cleavage in S-acylated thioester bonds using neutral hydroxylamine solution; and 3) biotinylation of the newly exposed thiol groups using sulfhydryl-reactive biotin such as N-[6-(biotinamido)hexyl]-3’-(2′-pyridyldithio) propionamide (biotin-HPDP) followed by streptavidin pulldown (Drisdel and Green, 2004; Drisdel et al., 2006; Wan et al., 2007). The enriched proteins of interest can subsequently be released from the biotin/streptavidin matrix with reducing agents. S-acylated proteins can then be detected by immunoblotting or identified by mass spectrometry.

As an alternative to biotin binding, the newly exposed protein thiol groups from step 2 after hydroxylamine treatment can be directly captured by sulfhydryl-reactive solid phase carriers (Forrester et al., 2011; Kumar et al., 2016) or magnetic microspheres conjugated to sulfhydryl-reactive compounds (Figure 1B) (Zhang et al., 2018). This alternative method is called Acyl resin-assisted capture (Acyl-RAC) assay. Interestingly, our group and others have found that ABE and Acyl-RAC are not always equally efficient in detecting all S-acylated proteins (Edmonds et al., 2017). Therefore, it is useful to try both methods when attempting to identify whether a protein-of-interest is S-acylated. In addition, PEG of various sizes can be used to react with free sulfhydryls generated in step 2 instead of biotin. This method is known as the acyl-PEG exchange assay (Howie et al., 2014; Percher et al., 2016). In this method, PEGylation gives a mass shift that can be detected by SDS-PAGE and western blot. Acyl-PEG exchange can not only quantify the number of acylation sites, but also show the relative amount of protein in each acylation state. In our experience, Acyl-PEG exchange is useful for some substrates but not for others. It is not known why some proteins are resistant to PEGylation at cysteine residues, but it may be related to steric constraints.

Acyl-group exchange methods can be applied to various protein samples, including cells (Chen et al., 2020) and tissues (Brigidi and Bamji, 2013) to detect S-acylation. They provide a higher specificity compared to metabolic labeling by limiting the capture to only hydroxylamine-sensitive thioester bonds, without capturing proteins from other functional groups. Perhaps most importantly, Acyl-group exchange detects proteins modified by endogenous lipids, which is a significant advantage over radiolabeling and click chemistry approaches. One significant disadvantage is that this method cannot discriminate what type of lipid was conjugated to the cysteine residue. It is also important to verify *bone fide* substrates using controls such as reactions without hydroxylamine treatment and mutagenesis to confirm the cysteine residue(s) being modified. Additionally, thioester bonds are also present in non-S-acylated proteins, including multiple members of the mitochondrial pyruvate dehydrogenase complex, the ubiquitin conjugating enzymes, and the active site intermediate of glyceraldehyde 3-phosphate dehydrogenase (GAPDH) (Roth et al., 2006; Wolfson-Stofko et al., 2013). Therefore, acyl-group exchange methods can lead to false positive results. It is also worth noting that acyl-group exchange methods provide snapshot-like profiles of S-acylated proteins at a given time point. Our group and others used these methods over a time course to evaluate the dynamic S-acylation (Chen et al., 2020; Shayahati et al., 2020).

### Proteomics

S-acylation proteomics can be achieved by performing metabolic labeling, ABE, or acyl-RAC followed by mass spectrometry. When using metabolic labeling, click chemistry is used to attach azide-conjugated biotin to the alkyne-conjugated lipid moieties incorporated onto proteins (Martin and Cravatt, 2009; Yount et al., 2010; Gao and Hannoush, 2014; Gao and Hannoush, 2014). Then acylated proteins can be enriched and analyzed using liquid chromatography and tandem mass spectrometry (Figure 1). Similarly in ABE or acyl-RAC procedures, proteins are eluted using reducing agents and analyzed using mass spectrometry. The relative abundance of proteins is estimated by using spectral counting (Old et al., 2005). Such approaches have been used to identify novel acylated protein substrates in different species and cell types (Roth et al., 2006; Martin and Cravatt, 2009; Nievas et al., 2018). By digesting the proteins at various steps before mass spectrometry, ABE or Acyl-RAC-based studies can also be used to identify the specific sites of acylation [reviewed in Wang and Yang (2021)]. Our group and others have successfully used proteomic approaches to compare the acylation proteome between samples, for example stimulated versus resting cells (Colquhoun et al., 2015; Sobocinska et al., 2018). With increasing evidence suggesting that S-acylation is highly dynamic, comparative proteomic studies can be especially useful in globally examining the temporal changes of S-acylation levels of signaling proteins.

One recently developed method to quantitatively compare the S-acylation profiles across two samples uses stable isotope labeling by amino acids in cell culture (SILAC) (Ong et al., 2002). Cells are first labeled with heavy or light isotope containing amino acids, mixed to minimize systemic errors, and then the acylated proteins from each sample are enriched using metabolic labeling (Martin et al., 2011) or ABE (Zhang et al., 2018). The heavy and light mass tags can be separated with mass spectrometry analysis and the relative abundance of S-acylation from two samples can be compared. SILAC has been used in several applications, including comparing the acylation proteomes of neuronal stem cells from wild-type and DHHC5 deficient mice, providing a list of 20 potential DHHC5 substrates (Li et al., 2012). In another study, two hepatocellular carcinoma cell lines with different metastasis potentials were examined and 151 proteins were identified to be differentially acylated (Zhang et al., 2018). Another improvement of SILAC was developed recently using three isotopes to allow more samples to be processed at the same time (Clark et al., 2016). This method can be potentially used to compare the acylation proteomes from more samples. Recently, ABE and stable isotope labeling in the whole animal (SILAM) were combined to look at Huntington’s disease (HD) by profiling S-acylation change in two mouse mutants (Wan et al., 2013). This approach shows promise in identifying the S-acylated proteome in animal models of human disease.

## Enzymatic Regulators of S-Acylation

### Palmitoyl-Acyl Transferases (PATs): From Yeast to Human

The enzymes that catalyze the transfer of acyl groups to cysteines on substrate proteins were first discovered in yeast (Lobo et al., 2002; Roth et al., 2002; Mitchell et al., 2006). Erf2, in the presence of Erf4, was described as a PAT that palmitoylates Ras (Bartels et al., 1999; Lobo et al., 2002). Later studies identified multiple proteins in this family, including the Huntingtin interacting protein 14 (HIP14) (Huang et al., 2004), Golgi-specific zinc finger protein (GODZ) (Keller et al., 2004), and Swf1 (Valdez-Taubas and Pelham, 2005). They share the conserved amino acid sequence D-H-H-C in a cysteine rich catalytic domain, leading to the nomenclature DHHC enzymes.

There are 23 PATs in human, which are encoded by the zinc-finger aspartate-histidine-histidine-cysteine (*ZDHHC*) genes 1 to 24 (there is no *ZDHHC10* gene) (Korycka et al., 2012). We will refer to these proteins as DHHC enzymes for the rest of this review. DHHC enzymes have tissue specific expression patterns (Fukata et al., 2006; Ohno et al., 2006). The majority of the DHHC enzymes localize to the Golgi membrane, however they can be retained in the ER or trafficked to the plasma membrane (Ohno et al., 2006). DHHC4 and DHHC6 have lysine-based sorting signals that retain them in ER membranes (Gorleku et al., 2011). DHHC2, 3, 5, 7, 8, 20, and 21 have been shown to traffic to the plasma membrane (Fernández-Hernando et al., 2006; Ohno et al., 2006; Akimzhanov and Boehning, 2015; Chen et al., 2019). All mammalian DHHC enzymes have at least four transmembrane domains with the DHHC motif in the cytosol [reviewed in Tabaczar et al. (2017)]. It is worth noting that the yeast PATs Akr1, Akr2, and Pfa5 have DHYC, and DHHC13 has DQHC at the conserved DHHC domain (Mitchell et al., 2006). However, mutation of the first His in Erf2 abolished its ability to acylate Ras (Lobo et al., 2002). Additionally, mutation of the Asp-His in Akr2 abolished its autoacylation as well as acylation of its substrate Yck2 (Roth et al., 2002). These studies indicate that the conserved DHHC motif is crucial for the enzymatic reaction.

Later studies found that autoacylation of the cysteine residue at the conserved DHHC domain serves as a reaction intermediate, suggesting a two-step ping-pong mechanism for DHHC enzymes (Mitchell et al., 2010; Jennings and Linder, 2012). Interestingly, the yeast PATs Swf1 and Pfa4 showed only partial loss of activity when the catalytic cysteine was mutated to arginine, suggesting the possibility of a direct nucleophilic attack on palmitoyl-CoA by the substrate (González Montoro et al., 2015). Despite the relatively conserved cysteine-rich domain around the catalytic core, whether individual PAT can have different enzymatic reaction mechanisms remains unclear.

The recently solved human DHHC20 and zebrafish DHHC15 crystal structures showed details about the organization of the DHHC active site (Rana et al., 2018). The cysteine-rich domain between the second and the third transmembrane domain associates with two zinc ions, which are important for positioning the active site. The aspartic acid and the histidines in the conserved DHHC motif position the cysteine for nucleophilic attack. The catalytic cysteine points toward the lipid bilayer, enabling the formation of reaction intermediate that inserts the acyl chain into the hydrophobic lipid bilayer. Another highly conserved motif in DHHC PATs is the Thr-Thr-x-Gly (TTxE) motif in the C-terminal tail right after the forth transmembrane domain (Mitchell et al., 2006). The TTxE motif is in close contact with the DHHC active site, and the second threonine forms a hydrogen bond with the aspartic acid of the active site (Rana et al., 2018). These features of the various conserved domains all contribute to the enzymatic reaction of DHHC proteins.

### Protein Substrate Recognition

All DHHC enzyme family members have distinctive N- and C-termini that are implicated in substrate recognition. There are several protein-protein interaction motifs unique to individual DHHC proteins. DHHC13 and 17 both have ankyrin repeats in the N-terminal region before the first transmembrane domain, and this domain functions to bind to the Huntingtin protein in neurons (Huang et al., 2004). When fusing the ankyrin repeats to the N-terminus of DHHC3, which is not a PAT for Huntingtin, the chimeric protein was able to palmitoylate Huntingtin and regulate its trafficking (Huang et al., 2009). Another example is the PSD-95/Discs-large/ZO-1 homology (PDZ) binding motif in the C-terminal tail of DHHC5/8. Binding and palmitoylation of the neuronal protein glutamate receptor interacting protein (GRIP1b) require the PDZ binding motif on DHHC5/8 (Thomas et al., 2012). This motif in also required for the interaction of DHHC5 with the synaptic scaffold protein PSD-95 (Brigidi et al., 2015). Not surprisingly, regions other than the well-characterized protein-protein interaction domains on DHHC enzymes can also be important for substrate recognition. In the very large 700 amino acid C-terminal tail of DHHC5, a 120 amino acid region right after the transmembrane domain, but not the PDZ domain, is crucial for recognition and palmitoylation of phospholemman (Howie et al., 2014).

Certain domains or amino acids on the substrates also affect recognition by specific DHHC enzymes. The yeast vacuolar protein Vac8 is palmitoylated by the yeast PAT Pfa3 in the N-terminal Src homology 4 (SH4) domain (Smotrys et al., 2005). Removing the C-terminal region after the SH4 domain of Vac8 enabled it to be palmitoylated by four additional PATs (Nadolski and Linder, 2009). In some cases, change in a single amino acid on the substrate protein can affect its recognition by DHHC enzymes. For example, the SNARE proteins SNAP25 and SNAP23 are structurally similar yet are not acylated by the same set of DHHC enzymes. SNAP25 can be S-acylated by DHHC15 but SNAP23 cannot. Mutation of Cys-79 of SNAP23 to mimic SNAP25 promoted its palmitoylation by DHHC15 (Greaves et al., 2010). The Cys mutation of SNAP23 may increase the direct interaction with DHHC15, or may promote membrane affinity and thus increase the likelihood of association with DHHC15. It is clear that the interaction between substrates and DHHC are regulated by various components. Therefore, studies mutating regions on both the DHHC enzyme and substrates for each substrate-DHHC pair will enable us to further understand PAT substrate recognition mechanisms. There is currently no way to predict which DHHC enzymes are responsible for the acylation of a specific protein. Recent advances in quantitative proteomic approaches can give us more answers by comparing cells with and without each of the DHHC enzymes.

### Lipid Substrate Specificity

DHHC proteins transfer the fatty acyl groups from acyl-coenzyme A (acyl-CoA) to protein substrates (Huang et al., 2004). The lipid substrate specificity was first studied for yeast Erf2/Erf4 complex by using unlabeled fatty acyl-CoA to compete with radiolabeled palmitoyl-CoA *in vitro* (Lobo et al., 2002). They found that shorter chain length such as lauryl (12-carbon) and myristoyl (14-carbon) showed weak inhibition, whereas 16 and 18-carbon saturated or unsaturated acyl-CoAs showed effective inhibition. Similar competition assay using 14–20-carbon saturated and unsaturated fatty acyl-CoAs were also used for studying DHHC2 and DHHC3 proteins (Jennings and Linder, 2012). It was found that all acyl-CoAs with longer than 14 carbons efficiently inhibited DHHC2 *in vitro* activity, whereas long chain fatty acyl-CoAs with 18 and 20 carbons showed minimal inhibition of DHHC3 activity. They also observed reduced rate of both autoacylation of DHHC3 and acyl group transfer to substrate when the 18-carbon stearoyl-CoA was used instead of the 16-carbon palmitoyl-CoA. These studies suggested that DHHC enzymes in general can incorporate a wide variety of fatty acyl chain lengths, but each enzyme has different degrees of lipid substrate selectivity. Extensive work has also been done to characterize the fatty acid selectivity in the highly similar DHHC3 and DHHC7 (Greaves et al., 2017). Through a series of domain swapping and point mutation experiments, one single amino acid in the third transmembrane domain of DHHC3 is shown to be crucial for its preference for shorter-chain fatty acids.

The crystal structures of human DHHC20 and zebrafish DHHC15 provided more insight into the mechanisms of lipid substrate selectivity (Rana et al., 2018). Both structures showed a hydrophobic cavity in which fatty acyl-CoA can be inserted into. All four transmembrane domains can interact with the acyl chain, and mutations of multiple highly conserved residues lining the cavity caused loss of enzymatic activity. In DHHC20, the tapered end of the cavity is sealed off by a hydrogen-bound between a Tyr and a Ser residue. Mutating the Tyr to the less bulky Ala caused an increased enzymatic activity using short-chain acyl-CoA. And mutating the Ser to the bulkier Phe shifted the preference toward long-chain acyl-CoA. These data suggested that the diversity of the amino acids lining the acyl chain binding cavity of different DHHC enzymes contribute to the differential lipid substrate selectivity observed (Rana et al., 2018).

### S-Acylation Cascades

Multiple DHHC enzymes have S-acylated residues outside of the autoacylated catalytic core. A proteomic study in a human prostate cancer cell line showed that DHHC5, 6, and 8 are acylated at three cysteines C-terminal to the DHHC catalytic core (Yang et al., 2010). It was proposed that S-acylation of DHHC5 causes conformational changes to allow substrate selection. Another study expanded the list of acylated DHHC enzymes to also include DHHC 16, 17, and 20 (Collins et al., 2017). In HeLa cells, acylation of the DHHC5 C-terminal tail is important for its interaction with substrate Golga7b, which subsequently stabilizes DHHC5 at the plasma membrane (Woodley and Collins, 2019). Our group found that in cardiomyocytes, the C-terminal tail of DHHC5 became acylated following beta-adrenergic stimulation (discussed in detail below) (Chen et al., 2020). It is worth noting that in our study, we found that the acylation of the DHHC5 C-terminal tail did not affect its autoacylation at the catalytic core. One recent study showed that DHHC20 can S-acylate the DHHC5 C-terminal tail and regulates its ability to interact with the Na pump (Plain et al., 2020).

One study reported a palmitoylation cascade in which DHHC16 palmitoylates three cysteines sites on the C-terminal tail of DHHC6 (Abrami et al., 2017). They found that the palmitoylation state of DHHC6 affected its degradation, localization within the ER membrane, and activity. The depalmitoylation of DHHC6 is catalyzed by APT2, which itself is acylated and activated by a yet-to-be-identified DHHC enzyme (Kong et al., 2013). These examples suggest that there are complex acylation/deacylation networks involving multiple DHHC and APT enzymes. The discoveries of acylation cascades marks one way by which we can start to understand the regulatory mechanisms of DHHC enzyme function. DHHC enzymes can also be regulated through post-translational modifications such as phosphorylation (Lievens et al., 2016), or their expression can be regulated through microRNAs (Chai et al., 2013). Therefore, when studying the S-acylation of a substrate protein, one must take into consideration the various levels of regulations contributing to the cellular functions and physiological effects.

### PATs and Human Diseases

Multiple DHHC enzymes have been implicated in human disease, including cancer (Table 2). DHHC2 expression is decreased in hepatocellular carcinoma (Peng et al., 2014) and gastric adenocarcinoma (Yan et al., 2013), and it acts as a tumor suppressor in metastasis. DHHC3 is shown to be upregulated in breast cancer, and increased expression correlates with decreased patient survival (Sharma et al., 2017). DHHC5 is suggested to drive oncogenesis in non-small cell lung cancer (Tian et al., 2015). Increased copy numbers of *zDHHC11* is implicated in bladder cancer (Yamamoto et al., 2007). DHHC14 overexpression is found in gastric cancer samples (Oo et al., 2014). DHHC enzymes and their substrates can affect the pathophysiology of cancer by regulating cell proliferation, cell migration, and apoptotic signaling [reviewed in Yeste-Velasco et al. (2015), Ko and Dixon (2018)]. For example, DHHC2 promotes cell-cell contact and inhibits epithelial-mesenchymal transition through its substrates CD9 and CD151, two cell surface proteins that regulate cell migration (Sharma et al., 2008). However, given the fact that DHHC2, like all other DHHC enzymes, has a variety of substrates, it is difficult to pinpoint the most critical substrates that contribute to cancer pathophysiology. This is one of several challenges in understanding how each DHHC enzymes contribute to disease pathogenesis.

**TABLE 2:** Summary of DHHC enzymes and human diseases. DHHC enzyme alterations in human diseases, including cancers and neurological diseases.

DHHC enzyme	Diseases	Alterations in DHHC	Related DHHC substrates	References
2	Gastric adenocarcinoma, Hepatocellular carcinoma	Reduced expression	CD9, CD151, CKAP4	175,176,183
3	Breast cancer	Up-regulated	ERGI3	177
5	Non-small cell lung cancer	Activated	Unidentified	178
8	Epilepsy, Schizophrenia	Increased expression, Genetic polymorphisms	Unidentified	184,185
9	X-linked intellectual disability	Loss of expression	Unidentified	186
11	Bladder cancer	Increased copy number	Unidentified	179
14	Gastric cancer, Leukemia	Increased expression, Activated	Unidentified	180
15	X-linked intellectual disability	Loss of expression	Unidentified	186
17	Glioblastoma	Up-regulated	H- and N- Ras	238

DHHC enzymes also contribute to neurological disorders. DHHC8 has increased expression in epilepsy patients (Yang et al., 2018), and single-nucleotide polymorphisms (SNPs) in the *zDHHC8* gene are associated with susceptibility to schizophrenia (Mukai et al., 2004). Mutations in *zDHHC9* are found in some families with X-linked mental retardation (Raymond et al., 2007). DHHC13 and 17 are known to palmitoylate the Huntingtin protein, and mouse models of DHHC13 and 17 show Huntington’s disease-like phenotypes (Yanai et al., 2006; Singaraja et al., 2011; Sutton et al., 2013). Many neuronal proteins/processes require protein acylation. DHHC9 promotes dendritic growth and synapse maturation. It has been shown that defective acylation of the small GTPases Ras and TC10 by DHHC9 contributes to dendritic dysfunction in animal knockout models (Shimell et al., 2019). The DHHC9 knockout mice also have seizure-like events suggesting that acylation by DHHC9 contributes to both intellectual disability and seizure-like disorders. The DHHC9 gene is located on the X-chromosome, and mutations associated with disease include insertions, frameshift mutations, and missense mutations suggesting mechanistically loss of function mutations predominate (Raymond et al., 2007; Mitchell et al., 2014). Female carriers are unaffected.

Many disease-associated proteins are known to require S-acylation for their functions. However, due to the redundancy of DHHC enzyme function and their various expression levels in certain tissues, the disruption of specific DHHC-substrate pairs will likely yield drastically different outcomes depending on the cell type. Therefore, more studies in cell lines and animal models are required to detail the DHHC-substrate pairs that contribute to disease pathophysiology.

## Enzymatic Regulators of Deacylation

### APT1 and APT2

Acyl protein thioesterase 1 (APT1) was purified from rat liver cytosol based upon its ability to deacylate H-Ras and Gαi *in vitro* (Duncan and Gilman, 1998). APT1 is a highly conserved α/β hydrolase, which contains both a G-X-S-X-G motif common in lipases and esterases and an active site S-H-D catalytic triad (Devedjiev et al., 2000). S-acylated proteins such as Gαi are the preferred substrates of APT1 *in vitro*. Consistent with its role as an acyl-protein thioesterase, overexpression of APT1 increases the basal turnover rate of S-acylated residues on many substrates, including small GTPases (Dekker et al., 2010; Kong et al., 2013), endothelial nitric oxide synthase (Yeh et al., 1999), and a number of other proteins. The neuronal microRNA miR-138 was found to modulate APT1 levels in synaptic spines, which reduce spine volume by decreasing Gα13 S-acylation (Siegel et al., 2009).

In addition to APT1, vertebrates express the paralogous enzyme APT2 (68% identity, 81% similarity) (Tomatis et al., 2010). APT1 and APT2 have different substrates. APT1 over-expression has no effect on growth associated protein 43 (GAP-43) S-acylation, whereas overexpression of APT2 promotes GAP-43 deacylation (Tomatis et al., 2010). In another example, only APT2 is essential for deacylation and stabilization of DHHC6 (Abrami et al., 2017), which is important for efficient S-acylation of the cytoplasmic domains of calnexin and multiple other S-acylated ER proteins. Moreover, agonist-dependent S-acylation of the β2-adrenergic receptor can only be reversed by APT1, but not APT2 (Adachi et al., 2016). It is still unclear why and how APT1 and APT2 prefer certain substrates *in vivo*. Peptide library screening *in vitro* indicates that amino acids surrounding the acylated residue may dictate preference for APT1 or APT2. However, the large overlap in substrate utilization between the two enzymes suggests that there is functional redundancy (Amara et al., 2019).

Perhaps not surprisingly based upon sequence homology, APT1 and APT2 share a lot of similarities in structure and function. Both APT1 and APT2 are members of the serine hydrolase family, which share a characteristic α/β-hydrolase fold and a hydrophobic cleft that accommodates the acyl chain (Devedjiev et al., 2000). They are broadly expressed in almost all tissues (Bachovchin et al., 2010; Abrami et al., 2017). It is worth noting that APT1 and 2 are primarily cytoplasmic (Yang et al., 2010), but can demonstrate partial internal membrane (Vartak et al., 2014) and plasma membrane (Kong et al., 2013) localization. Both enzymes have a cysteine at the second position after the initial methionine, which can be S-acylated to facilitate membrane localization (Kong et al., 2013; Vartak et al., 2014). APT1 and APT2 cycle on membranes through an acylation/deacylation cycle where auto-deacylation drives release from membranes into the cytosol compartment (Vartak et al., 2014).

We and others have shown that proteins that are acylated after cellular stimulation are deacylated just as rapidly (Akimzhanov and Boehning, 2015; Chen et al., 2019). This would be expected of a second messenger cascade. Interestingly, the kinetics of deacylation are substrate dependent. As an example, in cardiomyocytes Gα proteins are rapidly acylated and deacylated, while the inhibitory GPCR kinase GRK2 is acylated more slowly and persistently (Chen et al., 2019). Surprisingly, very little is known about the regulation of APT1 and APT2. In contrast to DHHC enzymes, the genetic diversity of APT enzymes is minimal (at least as we currently understand them). Both APT1 and APT2 are small (∼200aa) soluble proteins with no obvious structural motifs indicative of a regulatory mechanism. It is possible, however, to generate relatively specific chemical inhibitors despite high structural homology (Won et al., 2016), consistent with the observation of peptide substrate specificity (Amara et al., 2019). However, for S-acylation to be a *bone fide* signaling modification, regulation of the deacylation process should be a prerequisite. Interestingly, we found that APT1 was responsible for turnover of S-acylated groups on Lck under resting conditions, but not after cellular stimulation (Akimzhanov and Boehning, 2015).

### ABHD17 and Related Proteins

Two independent groups found that additional members of the α/β-hydrolase fold (ABHD) family of serine hydrolases are potent depalmitoylating enzymes for select substrates, including PSD-95 and N-Ras (Lin and Conibear, 2015; Yokoi et al., 2016). ABHD proteins and APTs both belong to the serine hydrolase superfamily and share extensive sequence homology (Long and Cravatt, 2011; Cao et al., 2019). Like APT enzymes, these enzymes are membrane anchored by palmitoylation. They have a unique pharmacological profile, and in particular are inhibited by hexadecylfluorophosphonate (HDFP) but not palmostatin B (Martin et al., 2012; Lin and Conibear, 2015). Deacylation activity against N-Ras was demonstrated for ABHD17A, ABHD17B, ABHD17C (Lin and Conibear, 2015). In addition to N-Ras, it is known that PSD-95 is rapidly deacylated in response to neuronal depolarization. To identify which enzyme deacylates PSD-95, a large functional overexpression screen to identify PSD-95 acyl-protein thioesterases was performed in cell lines (Yokoi et al., 2016; Tortosa et al., 2017). Of 37 tested proteins with putative thioesterase activity, ABHD12, 13, 17A, 17B, 17C, and APT1, 2 were shown to have significant activity for acylated PSD-95. The ABHD enzyme 17A, 17B, and 17C had the greatest activity in cell lines and neurons. Over-expression of ABHD17 enzymes reduced synaptic AMPA receptors and PSD-95 clustering in neurons, while the individual knockdown of ABHD17A/B/C stabilized S-acylated PSD-95 and inhibited PSD-95 clustering (Yokoi et al., 2016). Similar to APT1/2, ABHD17 enzymes themselves need to be S-acylated for plasma membrane association and to other potential S-acylated protein substrates. The cysteine rich domain in N-terminus is necessary for both S-acylation and plasma membrane association (Martin and Cravatt, 2009). Deletion of the cysteine rich domain in N-terminal has no effect on the activity of ABHD17, suggesting the S-acylation is only necessary for membrane localization but not for enzyme activity.

We are just beginning to understand the role of ABHD17 and related proteins as acyl-protein thioesterases, including structure, function, regulation, and substrate specificity. Recently in a global mutagenesis screen for suppressors of pulmonary metastasis, ABHD17A was identified as one of only eight metastatic suppressors (van der Weyden et al., 2017). The mechanisms by which it performs this function are unknown, but could presumably related to Ras or other oncogenic signaling pathways. Clearly more animal models are required to understand the physiology of this new class of acyl-protein thioesterase. Structural studies are also needed to understand if the N-terminal cysteine rich domain has functional roles in addition to acylation, such as a role in substrate engagement (Martin and Cravatt, 2009). Finally, almost nothing is known about the potential function of other ABHD enzymes that have shown *in vitro* acyl-protein thioesterase activity, including ABHD6, 12, 13 and 16A (Lin and Conibear, 2015; Yokoi et al., 2016). Recently ABHD10 was shown to localize exclusively in the mitochondria and deacylate the mitochondrial protein peroxiredoxin 5 (PRDX5), a key regulator of the antioxidation machinery (Cao et al., 2019). In addition to APT and ABHD proteins, there are lysosomal palmitoyl protein thioesterases 1 and 2 (PPT1/2), which are involved in protein degradation (Camp and Hofmann, 1993; Camp et al., 1994; Calero et al., 2003). PPTs are not thought to participate in S-acylation signaling events in the cytosol (Verkruyse and Hofmann, 1996), and therefore will not be discussed further in this review. It is worth noting that PPT1 deficiency is associated with neuronal ceroid lipofuscinosis, a severe neurodegenerative disease (Vesa et al., 1995). The functions of PPT proteins have been thoroughly reviewed elsewhere (Lu and Hofmann, 2006; Zeidman et al., 2009; Koster and Yoshii, 2019).

## Dynamic S-acylation/deacylation

Due to the reversible nature of protein S-acylation, the kinetics and the functional importance of dynamic acylation/deacylation cycles is a topic of great interest. Traditionally, pulse-chase experiments using radioactively labeled palmitate was used to determine the turnover of S-acylation, including G alpha proteins (Degtyarev et al., 1994) and Ras GTPases (Magee et al., 1987; Baker et al., 2003). However, evidence suggested that the pulse-chase method underestimated the turnover rates and is not sufficient for several reasons to determine the kinetics of cellular events that happens within seconds to minutes (Rocks et al., 2005). The understanding of dynamic S-acylation has been dramatically advanced by using more sensitive techniques, including click-chemistry-based metabolic labeling and acyl exchange methodologies described above. Studies by our group and many others showed that S-acylation is a rapid and reversible process necessary for cell signaling (Akimzhanov and Boehning, 2015; Fukata et al., 2016; Matt et al., 2019; Chen et al., 2020). These findings emphasize the importance of dynamic acylation in signal transduction similar to other post-translational modifications such as phosphorylation. Here we describe three examples of the dynamic acylation/deacylation cycle, the enzymatic regulators, and the cellular functions.

### G Alpha Proteins and Beta-Adrenergic Signaling

Beta-adrenergic receptors (β-ARs) are G protein-coupled transmembrane receptors that transmit extracellular signals to regulate contraction in the cardiomyocytes and other cell types. Unstimulated β-ARs are bound to heterotrimeric G-proteins, which consist of α, β, and γ subunits. Ligand binding to β-ARs leads to the exchange of GDP for GTP on the Gα subunit, and as such the receptor acts as a guanine nucleotide exchange factor. Activated Gα and Gβγ subunits then dissociate and act upon downstream signaling pathways. In the heart, β-AR signaling has an important role in regulating contraction. Cardiac β-ARs interact with both Gαs and Gαi. Several members in β-AR signaling pathway are S-acylated in cardiomyocytes, such as β2-AR, Gαs, Gαi, and adenylyl cyclase 5/6 (Rybin et al., 2000; Ostrom et al., 2001; Steinberg and Brunton, 2001), and S-acylation of these proteins is crucial for subsequent functions (Thinon et al., 2016; Wedegaertner et al., 1993). The two major sub-types of β-ARs in mammalian hearts are the β1-AR and β2-AR. Both receptors interact with Gαs, leading to downstream adenylyl cyclase activation and production of cAMP. Acylation of β-AR signaling proteins can lead to enrichment in caveolae, where signaling components assemble as a macromolecular signaling complex (Chen et al., 2020; Essandoh et al., 2020). Caveolae are specialized lipid rafts/membrane microdomains, and function in part to enrich S-acylated proteins important for cellular signaling in the heart (Gratton et al., 2004; Balijepalli et al., 2008).

**FIGURE 2:**
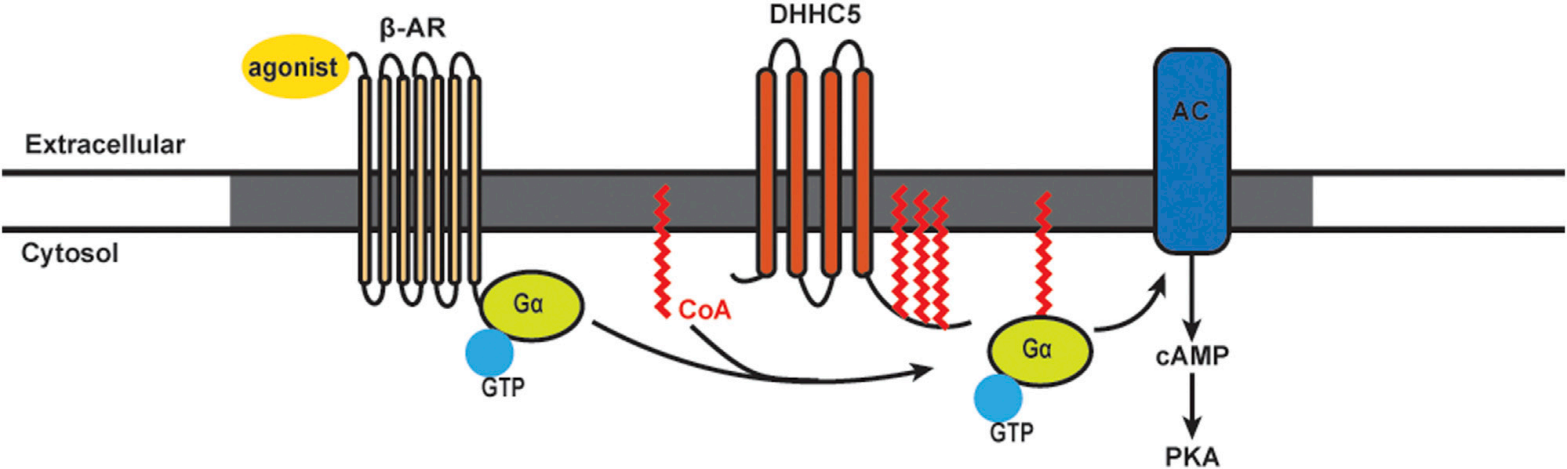
Schematics of S-acylation of Gα protein and β-AR signaling pathway. Upon agonist stimulation, β-adrenergic receptors (β-AR) bind DHHC5 palmitoylated Gα and leads to the activation of adenylyl cyclase (AC) and production of cAMP, followed by activation of Protein Kinase A (PKA). β-AR, AC and certain PKA subunits are enriched in caveolae (grey).

It has been known for many years that β-AR stimulation leads to rapid turnover of the S-acylated groups on Gαs. The model for stimulus-dependent acylation was based on the availability of free Gαs: separation from the βγ subunits allowed the access of putative S-acylation/deacylation enzymes (Wedegaertner et al., 1993; Degtyarev et al., 1993). We recently showed an alternative mechanism for Gα acylation that requires stimulus-dependent plasma membrane DHHC5 activation (Chen et al., 2020). The β2-AR itself is acylated on cysteine 341. Mutation of this residue results in reduced capacity to couple to adenylyl cyclase and alterations in phosphorylation, trafficking, and recruitment of β-arrestin 2 and phosphodiesterase (PDE) 4D (Liu et al., 2012; O'Dowd et al., 1989; Moffett et al., 1996). Ultimately, inhibiting β2-AR acylation leads to an increased rate of cardiomyocyte contraction (Liu et al., 2012). It has also been shown that Golgi-resident DHHC9/14/18 are redundantly responsible for S-acylation of the β2-AR on cysteine 265, and APT1 is the enzyme for β2-AR deacylation (Adachi et al., 2016). S-acylated β2-ARs are retained at the plasma membrane, and under conditions of sustained adrenergic stimulation they are resistant to internalization until deacylated by APT1 (Adachi et al., 2016). For S-acylation of Gα proteins, it has been shown that DHHC3 and DHHC7 can S-acylate Gαq, Gαs, and Gαi2 in HEK293T cells (Tsutsumi et al., 2009). However, another study found that stimulation of β-ARs in cell lines did not induce detectable changes in Gα protein S-acylation levels (Jones et al., 1997). More recently, our lab found S-acylation levels of both Gαs and Gαi were increased rapidly following β-AR stimulation [Figure 2; Chen et al. (2020)]. By 30 min, the S-acylation levels of Gαs and Gαi were returned to baseline, presumably indicating activation of acyl-protein thioesterases. The agonist-induced S-acylation was required for downstream cAMP production and contractile responses (Chen et al., 2020). Together, there is very strong evidence that stimulus-dependent S-acylation is an essential regulator of β-AR signaling and downstream modulation of heart contraction.

The DHHC5 enzyme is localized on the cardiomyocytes plasma membrane and enriched in caveolae. DHHC5 can regulate the dynamic S-acylation of phospholemman, a regulatory subunit of the Na pump, in a stimulus-dependent manner (Tulloch et al., 2011; Howie et al., 2014). Knocking down DHHC5 in cardiomyocytes showed that this enzyme is essential for S-acylation of Gαs, Gαi, and downstream functional responses of β-AR stimulation (Chen et al., 2020). Using purified components, an S-acylation assay showed that Gαs and Gαi are direct substrates of DHHC5. DHHC5 itself is subject to dynamic S-acylation, and like the β-AR, this modification stabilizes the enzyme at the plasma membrane. Importantly, S-acylation of the DHHC5 carboxy-terminal tail is required for cAMP production and downstream contractile responses in cultures cardiomyocytes (Chen et al., 2020).

In addition to *in vitro* models, the functional significance of DHHC5 in regulating cardiac function has been investigated in a DHHC5 gene trap mouse model (Li et al., 2012; Lin et al., 2013). Ischemia/reperfusion activates massive endocytosis (also called MEND) in cardiomyocytes. DHHC5 deficiency significantly reduces MEND upon reoxygenation, which improves cardiac performance. The substrates by which DHHC5 promotes MEND and sarcolemmal turnover are proposed to be the Na/K pump, the Na pump regulator phospholemman, flotillin-2, and other myocyte membrane proteins (Lin et al., 2013). It was concluded that DHHC5 regulates contractility by modulating sarcolemmal turnover, and this process is detrimental during anoxic stress. It would be interesting in future studies to compare cardiac function *in vivo* in wild-type and gene trap DHHC5 mice under normal and cardiac stress conditions.

### Lck and T Cell Signaling

**FIGURE 3:**
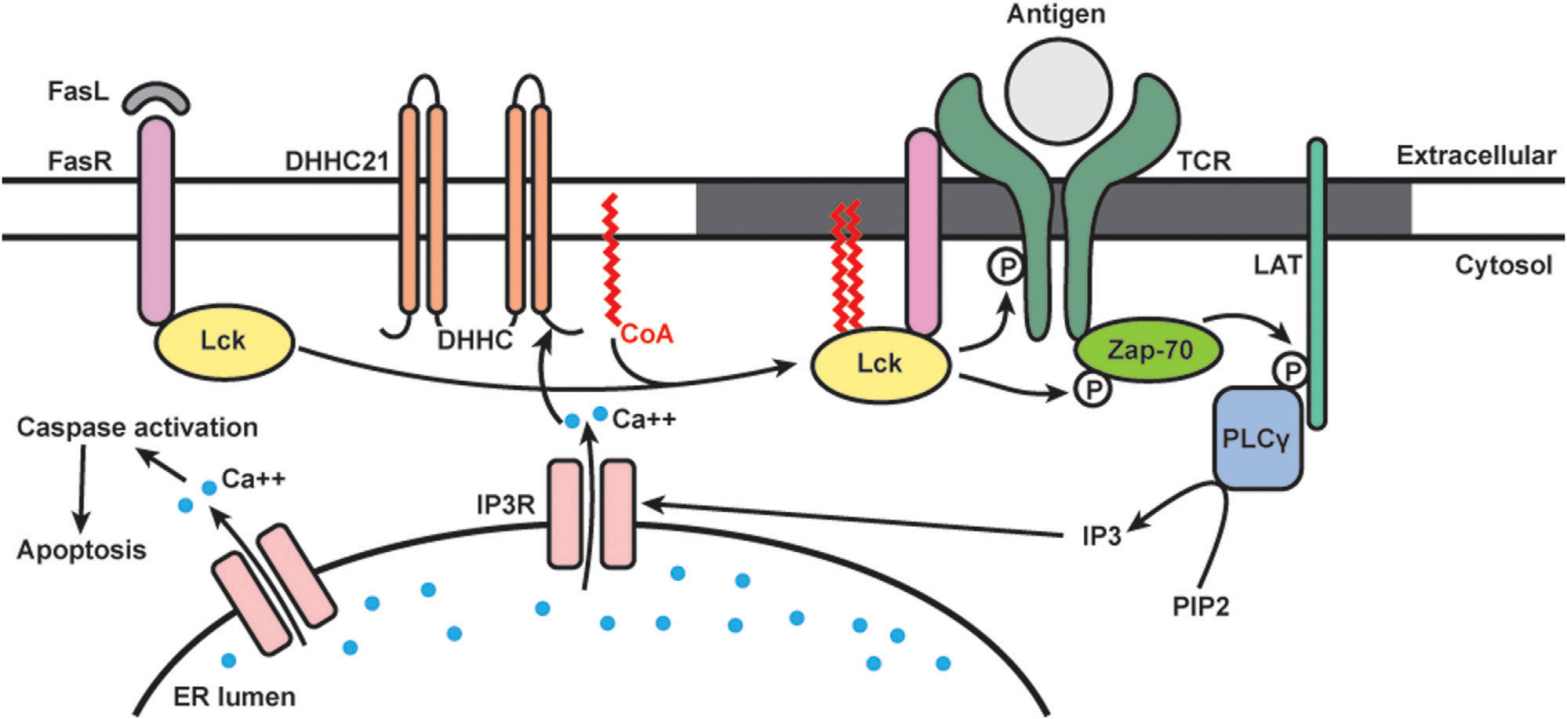
Schematics of dynamic S-acylation of Lck in T cells. Upon Fas receptor (FasR) activation by Fas ligand (FasL), DHHC21 palmitoylates Lck, which localizes it to lipid raft (grey) to activate the TCR signaling complex. Downstream calcium (blue) release from ER leads to apoptosis.

The Fas receptor (FasR) is a member of the tumor necrosis factor receptor family, and upon activation by Fas ligand, it triggers apoptotic cell death (Nagata, 1997). In T cells, repeated T-cell receptor (TCR) stimulation and the binding of Fas ligand to the receptor leads to activation-induced cell death, a process crucial for the removal of reactive T cells after the invading antigens are eliminated (Green et al., 2003). Fas signaling in T cells involves multiple members of the canonical TCR signaling complex (Akimzhanov et al., 2010). Upon activation, Src kinases Lck and Fyn are recruited and phosphorylate the cytoplasmic tail of TCR, which subsequently recruits zeta chain-associated protein 70 kDa (ZAP-70). ZAP-70 phosphorylates the adaptor protein linker for the activation of T cells (LAT). Activated LAT recruits a series of signaling proteins including phospholipase C-γ1 (PLCγ1), which hydrolyzes phosphatidylinositol bisphosphate [Figure 3, reviewed in Huse (2009)]. Our group found that activation of Lck and TCR signaling is required for the production of secondary messenger inositol 1,4,5-triphosphate (IP_3_) during Fas-mediated apoptosis in T cells (Akimzhanov et al., 2010).

Many members of the TCR signaling pathway are known S-acylated proteins. Lck is dually palmitoylated at Cys-3 and 5, and mutation of these sites prevents Lck from activating downstream TCR signaling (Paige et al., 1993; Yurchak and Sefton, 1995; Kabouridis et al., 1997). Our group is interested in studying Lck S-acylation and its functional roles in Fas signaling (Figure 3). We found that in T cells, S-acylation of both cysteines on Lck is required for Fas-mediated apoptotic events, including calcium release, caspase-3 activation, and cell death (Akimzhanov and Boehning, 2015). By using metabolic labeling, we analyzed the palmitic turnover kinetics on Lck and Fyn. We found that Lck has faster turnover rate (in minutes) than Fyn (in hours), suggesting that they are likely regulated by different enzymatic mechanisms despite very high homology in both structure and function. Notably, Lck has increased *de novo* palmitoylation within 2 min of Fas ligand stimulation, and the rate of palmitoylation decreased after 10 min. This rapid and transient Lck palmitoylation kinetically correlates to activation of downstream events, including ZAP-70 and PLCγ1 activation.

To study the enzymatic machineries regulating the rapid, stimulus-dependent Lck palmitoylation, we focused on plasma membrane localized DHHC enzymes. We identified DHHC21 as the enzyme responsible for rapid Lck palmitoylation and downstream Fas signaling in T cells. We also found that the rapid palmitoylation of Lck following Fas stimulation is dependent on intracellular calcium levels. This is likely mediated by a calcium/calmodulin binding site in the C-terminal tail of DHHC21 (Shayahati et al., 2020). Interestingly, we found that though APT1 deacylates Lck under resting conditions, it does not affect stimulus-dependent deacylation following Fas stimulation.

Multiple proteins in the Fas signaling pathways are known to require S-acylation for proper localization and downstream signaling. Fas receptor is palmitoylated by DHHC7 and recruited to lipid rafts to form stable oligomers important for the formation of the death-induced signaling complex (Chakrabandhu et al., 2007; Rossin et al., 2015). Both Lck and LAT palmitoylation lead to increased lipid raft affinity and ensure activation of TCR signaling cascade (Kabouridis et al., 1997; Zhang et al., 1998; Lin et al., 1999). However, most of these studies utilized cysteine mutant proteins and thus cannot reflect the dynamic acylation/deacylation cycles happening at the plasma membrane. Our study supports the well-known model in which lipid rafts serve as platforms for the assembly of TCR signaling proteins (Janes et al., 2000; Kabouridis, 2006; He and Marguet, 2008). Specifically, our study showed that the rapid, agonist-induced acylation and deacylation kinetics is temporally consistent with proximal signaling events. It is also worth noting that DHHC and APT proteins are identified as new classes of regulators of T-cell signaling. More recently, we have shown that DHHC21 is a critical regulator of T cell development/differentiation *in vivo* using the *depilated* mouse model (Lin et al., 2013).

### N and H Ras Signaling

The Ras family are small GTPases involved in cell signaling, proliferation, differentiation, and survival (Hancock, 2003; Castellano and Santos, 2011). In humans there are four Ras proteins: N-Ras, H-Ras, and two K-Ras variants (Hancock, 2003; Castellano and Santos, 2011; Cox et al., 2014; Hobbs et al., 2016). Traditionally, Ras was considered only to be active on the plasma membrane (Apolloni et al., 2000). Stimuli such as Son of Sevenless (SOS) or epidermal growth factor (EGF) activates Ras *via* the exchange of GDP for GTP on the protein (Bandaru et al., 2019). Subsequently, Ras-GTP recruits the serine/threonine kinase Raf-1 to the plasma membrane (Stokoe et al., 1994). Active Raf-1 then activates mitogen-activated protein kinase (MAPK), which phosphorylates and activates downstream substrates (Mor and Philips, 2006). Besides Raf-1 and its homologs, the best-studied effector of Ras is phosphatidylinositol 3-kinase (PI3K) (Mor and Philips, 2006). PI3K activates Akt and c-Jun N-terminal kinases (JNK) to promotes cell survival (Mor and Philips, 2006).

**FIGURE 4:**
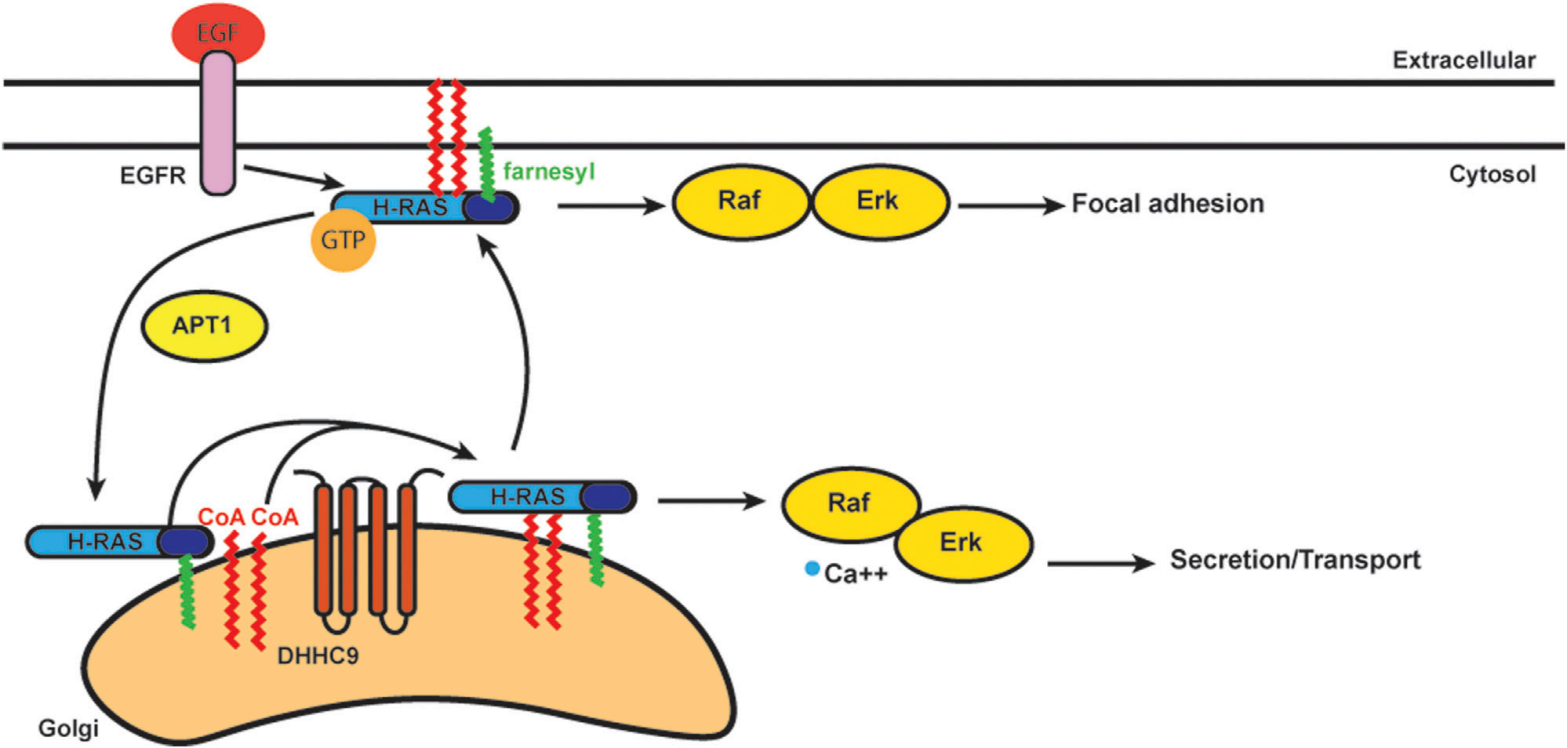
Schematics of acylation/deacylation regulation of H-RAS localization and signaling. DHHC9 S-acylates H-RAS to stablize its localization on plasma membrance (PM) and activates the Raf-Erk pathway to regulate focal adhesion.Once the S-acylation is removed by APT1, H-RAS that is only farnesylated does not bind PM and cycles to Golgi.

S-acylation is known to stabilize N-Ras and H-Ras at the plasma membrane (Figure 4) (Laude and Prior, 2008). S-acylation deficient N-Ras can still bind GTP, but cannot localize to the plasma membrane and loses the ability to activate both MAPK and PI3K downstream signaling pathways involved in cellular transformation (Cuiffo and Ren, 2010). Stimulation of growth factor receptors normally induces a rapid but transient activation of H-Ras at the plasma membrane and delayed but long-lasting activity at the Golgi (Chiu et al., 2002; Rocks et al., 2005). Deacylation inhibitors have no effect on this EGF-induced transient increase of H-Ras-GTP on the plasma membrane, but they decrease the H-Ras-GTP pool on the Golgi. The activity of H-Ras on the Golgi also depends largely on retrograde transport of the activated protein from plasma membrane through the S-acylation cycle (Rocks et al., 2005).

In addition to the plasma membrane and Golgi, Ras is also activated on ER membranes (Chiu et al., 2002). In order to study how Ras localization regulates cell growth, S-acylation deficient H-Ras was specifically targeted to the Golgi, ER and plasma membrane. Ras located at the Golgi complex was the least efficient at activating Akt, while ER and Golgi were the preferred sites by Ras to activate JNK (Matallanas et al., 2006). The Ras pools present at plasma membrane were considerably less effective for activating Akt or JNK, but efficiently activated Erk. Ras localized to the Golgi induced slow growth of fibroblasts, while Ras localized in plasma membrane induced cell transformation (Matallanas et al., 2006). Thus, S-acylation plays an important role in Ras localization and trafficking with implications for signaling and cell transformation.

As mentioned, trafficking of Ras from the Golgi to the plasma membrane requires S-acylation and proceeds *via* vesicular transport (Choy et al., 1999; Apolloni et al., 2000). N-Ras and H-Ras are S-acylated at one (N-Ras) or two (H-Ras) cysteines by DHHC9-GPC16 at the Golgi apparatus and transported to the plasma membrane (Swarthout et al., 2005). Upon deacylation they transport back to the Golgi to complete the Golgi/plasma membrane transport cycle (Lin et al., 2017). Blocking deacylation prolongs plasma membrane association of Ras and dysregulates the endomembrane system (Dekker et al., 2010; Rocks et al., 2010). APT1 was identified for its *in vitro* deacylation of H-Ras (Duncan and Gilman, 1998). ABHD17, as mentioned above, can deacylate N-Ras (Lin and Conibear, 2015). Palmitate turnover of N-Ras was significantly inhibited when all three ABHD17 members were downregulated simultaneously. The number of S-acylation sites can also affect the half-life of N-Ras and H-Ras on the plasma membrane. N-Ras has only one S-acylation site, and its half-life is much shorter than H-Ras, which has two S-acylation sites (Rocks et al., 2005).

## Conclusion

In this review, we summarized key findings in the field of protein S-acylation and the enzymatic regulators. Much of the field is now focusing on dynamic acylation/deacylation cycles and the role of this process in cell signaling. A significant gap in our understanding is how DHHC protein acyltransferases and acyl-protein thioesterases are regulated in a stimulus-dependent manner. This will be essential for understanding how they regulate cell signaling and the potential for therapeutic intervention. Another major question is the mechanisms by which DHHC and APT/ABHD proteins determine substrate specificity. Despite the advent of large-scale unbiased peptide substrate screens and high resolution structures, this is still a poorly understood aspect of the acylation machinery.

A number of pharmacological tools have been developed to mediate S-acylation reactions. The most widely used inhibitor of S-acylation is 2-bromopalmitate (2BP), a non-metabolizable analogue of palmitate (Webb et al., 2000). 2BP and other lipid-based compounds such as tunicamycin (Patterson and Skene, 1995) and cerulenin (DeJesus and Bizzozero, 2002) non-selectively inhibits the incorporation of fatty acyl groups onto target proteins. However, they are known to be highly toxic to cultured cells and target many proteins in the cellular lipid metabolism pathways, making them poor tools to study S-acylation (reviewed in Draper and Smith (2009), Chavda et al. (2014)]. Given the increasing interest in targeting protein S-acylation in various diseases, several chemical screens have been conducted to identify better inhibitors of DHHC enzymes or specific S-acylation substrates (Ducker et al., 2006; Hamel et al., 2016; Ganesan et al., 2017). Further studies are needed to define the mechanisms of actions for the compounds identified in these screens. In the future we anticipate the development of novel chemical tools with specific utility in studying highly dynamic and transient acylation/deacylation cycles. As an example, a new methodology for site-specific incorporation of lipids at cycloalkyne-conjugated unnatural amino acids was recently described which should facilitate understanding the attachment of specific lipids at defined sites “on demand” (Li et al., 2020).

We have a nascent understanding of the functional outcomes of the attachment of lipids other than the 16-carbon saturated fatty acid palmitic acid. Future work will need to address the functional outcomes and prevalence of S-acylation with other chain lengths or unsaturated fatty acids. As an example, S-acylation with unsaturated lipids such as arachidonic acid would be predicted to exclude substrates from rafts, which could be an important regulatory mechanism for the inclusion or exclusion of S-acylated proteins from rafts. Finally, the field would benefit with more animal/*in vivo* models. The function of the broad diversity of protein acyltransferases and the ever expanding family of acyl-protein thioesterases is still very poorly understood *in vivo*.
